# Silver and Zinc Oxide Nanoparticles for Effective Aquaculture Wastewater Treatment

**DOI:** 10.3390/nano15070559

**Published:** 2025-04-05

**Authors:** Mahmoud Abou-Okada, Mansour El-Matbouli, Mona Saleh

**Affiliations:** 1 Division of Fish Health, University of Veterinary Medicine, 1210 Vienna, Austria; abouokada.mm@cu.edu.eg (M.A.-O.); mansour.el-matbouli@vetmeduni.ac.at (M.E.-M.); 2 Aquatic Animal Medicine and Management, Faculty of Veterinary Medicine, Cairo University, Giza 12211, Egypt

**Keywords:** nanomaterials, wastewater, ammonia nitrogen, microbial load, zinc concentrations

## Abstract

This study explores the use of silver nanoparticles (Ag NPs) and zinc oxide nanoparticles (ZnO NPs), either singly or in combination, for the nanoremediation of aquaculture wastewater. Aquaculture wastewater was treated with varying doses of Ag NPs and ZnO NPs across the following six groups: Group 1 (0.05 mg Ag NPs/L), Group 2 (1 mg ZnO NPs/L), Group 3 (0.05 mg Ag NPs/L + 1 mg ZnO NPs/L), Group 4 (0.025 Ag NPs/L + 0.5 mg ZnO NPs/L), Group 5 (0.1 mg Ag NPs/L + 2 mg ZnO NPs/L), and a control group. Water quality, microbial loads and nanomaterial concentrations were assessed over ten days. Transmission electron microscopy (TEM) showed average particle sizes of 102.5 nm for Ag NPs and 110.27 nm for ZnO NPs. The removal efficiencies of NH_4_-N were over 98% across treatment groups. In addition, COD removal efficiencies were 33.33%, 68.82%, 49.59%, 61.49%, and 37.65%. The log-reductions in aerobic plate counts for the nanoparticle-treated wastewater were 1.191, 1.947, 1.133, 1.071, and 0.087, compared to a reduction of 0.911 in untreated wastewater. Silver concentrations ranged from 0.0079 to 0.0192 mg/L, while zinc concentrations ranged from 0.3040 to 0.9740 mg/L, indicating that ZnO-NPs represent a sustainable treatment method for aquaculture wastewater.

## 1. Introduction

The One Health approach in aquatic ecosystems highlights the connection between human health, fish health, and environmental health [1]. The growing demand for accessible water, coupled with the issue of polluted organic waste in water bodies, poses a critical challenge to sustainable development. From the One Health and sustainability prospectives, preservation of aquatic ecosystems is one of the sustainable development goals (SDGs) of the United Nations [2]. Aquaculture, one of the fastest growing and most highly traded food sectors worldwide, is expected to provide most of the world’s aquatic protein by 2050 [3]. However, there are concerns about the environmental impacts of aquaculture effluents on the receiving ecosystems. Despite these criticisms, the inherent benefits of aquaculture, including massive food production and economical profits, have led the researchers to seek diverse sustainable strategies to mitigate the negative impacts rather than just prohibiting this activity [4].

Unsustainable aquaculture practices include the use of commercial feeds, excessive use of chemicals and drugs, excessive use of organic and inorganic fertilizers, accumulation of fish metabolic wastes, in addition to the decomposition of dead fish and uneaten feed, which result in the pollution of aquatic ecosystems [5,6]. Aquaculture effluents are an important point source of pollution, particularly for nitrogenous wastes (total ammonia nitrogen, nitrite and nitrate), total phosphorus, chemical oxygen demand (COD) and pathogens, which have significant adverse effects on the water quality and, in turn, jeopardize the survival of aquatic organisms in natural water bodies [5,7].

Dissolved waste is the result of the metabolic processes of fish or decomposed, uneaten feed. The two major components of concern in these dissolved wastes are nitrogen (N) and phosphorus (P) [8]. Ammonia is a critical pollutant of fish culture water, especially in the un-ionized form (NH_3_). Ammonia (NH_3_) is highly toxic to the fish cultured in the system and those in receiving water bodies, if not treated before released into the environment [9]. Phosphorus is another important metabolite or decomposed product of aquaculture feed that is also poorly utilized. Unlike ammonia, phosphorus is not toxic to cultured fish, but when discharged to the environment, it mixes into enriches natural water bodies and may contribute to eutrophication, depending on its concentration, frequency of release, and the size of the receiving water body [7,10].

Aquaculture effluents are released into natural water bodies; thus, the removal of these pollutants through environmentally friendly and efficient methods is crucial. Nanomaterials are mainly used to overcome major water and wastewater problems. The term “nanomaterial” refers to materials of the nano scale, with a nanometer being a trillionth of a meter in size [11,12]. The use of nanoparticles to reduce contaminants has grown in recent decades. Their small size, high reactivity, and catalytic properties make nanoparticles effective candidates for remediating contaminated water [13]. Nanoremediation is an innovative remediation technique that relies on the use of nanomaterials. Nanoremediation approaches can provide sustainable solutions to the environmental pollution problems and could depreciate the financial burden for clean-up of contaminated sites [14,15].

Silver nanoparticles (Ag NPs) have been proven to be a good antimicrobial agent, with wide applications for water disinfection. In addition to this, treatment with Ag NPs decreased the diversity of microbial communities without significantly affecting their functioning [16,17]. The modes of action of Ag NPs include their interaction with the bacterial cell wall, the generation of reactive oxygen species (ROS), interaction with DNA, and release of Ag^+^ ions [18,19]. Zinc Oxide nanoparticles (ZnO NPs) are regarded as a good photocatalyst due to their high electrochemical stability, super oxidative capability, excellent biocompatibility, low cost and low toxicity. Due to their specific properties, ZnO NPs are considered one of the most promising candidates for catalytic water treatment. Moreover, ZnO NPs are stable under harsh processing conditions, which make them suitable for various antimicrobial applications [17,20]. The ZnO nanoparticles exhibit potent antimicrobial properties through complex mechanisms of action, which involve ROS formation, liberation of antimicrobial ions mainly Zn^2+^ ions, electrostatic interactions and internalization of ZnO NPs into bacteria resulting in inhibition of cellular processes like glycolysis, acid tolerance, and transmembrane proton translocation [21,22].

The use of metallic nanoparticles (MNPs) to treat aquaculture wastewater has received relatively little attention, particularly in the application of nanoparticles either singly or in combination as nanoremediation of aquaculture wastewater. Many previous studies have highlighted the use of metallic nanoparticles to treat wastewater effluents in wastewater treatment plants (WWTPs) [16,23,24,25,26].

The widespread use of metallic nanoparticles (MNPs) often results in their release into the environment, particularly from wastewater treatment plants (WWTPs). During the wastewater treatment processes, nanoparticles can undergo various transformations, including adsorption, stabilization/aggregation, dissolution and surface transformation. These processes may lead to increased concentrations of nanoparticles in both effluents and sludge [27]. While most Ag NPs tend to accumulate in the sludge, a significant portion is still expected to be released into the effluent [28]. The exposure of aquatic ecosystems to high concentrations of Ag NPs and ZnO NPs can alter the physicochemical and biological properties of water, affecting aquatic organisms and potentially leading to significant ecotoxicological impacts [29].

Aquaculture wastewater treatment remains a critical challenge due to the accumulation of pollutants such as nutrients, organic matter, and microbial contaminants. While conventional remediation methods exist, their efficiency and sustainability limitations highlight the need for innovative solutions. This study addresses this gap by evaluating the effectiveness of silver nanoparticles (Ag NPs) and zinc oxide nanoparticles (ZnO NPs), applied individually or in combination, for the remediation of aquaculture wastewater. Specifically, the research aims to (1) characterize the physicochemical and microbial profile of aquaculture wastewater, and (2) assess the dose-dependent efficacy of Ag NPs and ZnO NPs in improving water quality. Key parameters monitored include ammonia nitrogen (NH_4_-N), total phosphorus (PO_4_-P), nitrite nitrogen (NO_2_-N), nitrate nitrogen (NO_3_-N), chemical oxygen demand (COD), microbial loads, and residual nanoparticle concentrations. By systematically analyzing these factors, the study seeks to provide actionable insights into nanoparticle-based strategies for sustainable wastewater management in aquaculture systems.

## 2. Materials and Methods

### 2.1. Materials

Silver nanoparticle (Ag NP) dispersion (0.02 mg/L in aqueous buffer, 100 nm particle size (TEM) and sodium citrate as stabilizer), non-capped zinc oxide nanoparticle (ZnO NPs) dispersion (50% wt. in H_2_O, 100 nm particle size (TEM), and Ag NPs and ZnO NPs were obtained from Sigma-Aldrich chemical, GmbH, Germany. All chemicals were analytical reagents, and were used without further purification. For the preparation of aqueous solutions, double-distilled and deionized water with a Milli-Q water purification system (Millipore, Darmstadt, Germany, E-POD™, 18.2 MΩ cm^−1^ @ 25 °C) was used throughout the study.

The water analysis was performed using Hach Lange test kits (Hach Lange, GmbH, Düsseldorf, Germany), with calibration procedures and detection limits as follows: For ammonium (NH_4_-N), LCK 304 and LCK 505 kits were used, covering a range of 0.015–5 mg/L. Similarly, nitrite (NO_2_-N) was measured using LCK 341 and LCK 342 (0.015–6 mg/L), while nitrate (NO_3_-N) was analyzed via LCK 339 and LCK 340 (0.23–35 mg/L). Additionally, total phosphorus (PO_4_-P) was quantified using LCK 348 and LCK 349 (0.05–5 mg/L), and chemical oxygen demand (COD) was determined with LCK 1414 (5–60 mg/L). Silver (Ag) concentrations were quantified using the LCK 354 test kit, with a measurable range of 0.04–0.8 mg/L, while zinc (Zn) levels were determined using LCK 360 and LCS 360 kits (Hach Lange, GmbH, Düsseldorf, Germany), covering a range of 0.02–6 mg/L (Figures S1 and S2). To enhance measurement accuracy, particularly for silver concentrations, we additionally employed the HI-97737 silver photometer (Hanna Instruments, GmbH, Vöhringen, Germany), which offers a more sensitive detection range of 0.000–1.000 mg/L Ag (Figure S2).

### 2.2. Characterization of Nanoparticles

The particle size distribution of the nanoparticles was analyzed using Dynamic Light Scattering (DLS) with ZetaSizer NanoZS (Malvern Panalytical, Malvern, UK). To examine the particle size and shape, transmission electron microscopy (TEM; EM900, Zeiss, Oberkochen, Germany) was used, operating at an acceleration voltage of 80 kV, with a tungsten hairpin cathode and a wide-angle dual speed 2k CCD camera (EMSIS GmbH, Münster, Germany). A twenty-five-microliter sample of nanoparticle solution was placed onto graphite-coated copper grids and allowed to dry overnight before imaging. The average particle size was derived from at least 50 randomly sampled nanoparticles using ImageJ^®^ software version 1.54p. Zeta potential was measured using the ZetaSizer NanoZS (Malvern Panalytical, Malvern, UK).

The absorption spectra were determined using a UV–Vis spectrophotometer (NanoDrop 2000^®^, Thermo Fischer Scientific, Waltham, MA, USA) against a blank (deionized water). ZnO NPs were sonicated for 10 min. before measurement. The spectra were observed over a wavelength range of 200–700 nm. All measurements were conducted at room temperature across three separate days.

### 2.3. Wastewater Characterization

The aquaculture wastewater was obtained from fiberglass aquaria of Fish Health Division, University of Veterinary Medicine, Vienna, Austria. The aquaculture wastewater samples were subjected to physical, chemical and microbiological analyses.

### 2.4. Experimental Design

Twelve identical glass tanks (45 × 40 × 50 cm, 15 L working volume) were arranged in a controlled environment chamber. Each tank was equipped with fine-bubble airstones for consistent aeration, a digital temperature controller (±0.1 °C accuracy), and light timers for photoperiod regulation. The experimental design included six treatment groups, each replicated twice (*n* = 2), consisting of the following: an untreated wastewater control (Control); single nanoparticle treatments with either 0.05 mg/L silver nanoparticles (Group 1—Ag NPs) or 1 mg/L zinc oxide nanoparticles (Group 2—ZnO NPs); and three combination treatments with varying concentrations—0.05 mg/L Ag NPs plus 1 mg/L ZnO NPs (Group 3, Ag NPs + ZnO NPs), half-strength doses at 0.025 mg/L Ag NPs plus 0.5 mg/L ZnO NPs (Group 4, half dose), and double-strength doses at 0.1 mg/L Ag NPs plus 2 mg/L ZnO NPs (Group 5, double dose).

Prior to experimentation, all glass tanks underwent rigorous pre-treatment conditioning, including acid washing and triple rinsing with deionized water to eliminate contaminants. Freshly prepared nanoparticle stock solutions were sonicated for 30 min at 40 kHz to ensure proper dispersion before dosing. The systems were allowed to stabilize for 24 h with nanoparticles prior to wastewater introduction to establish equilibrium conditions. The dosing protocol consisted of an initial 24 h pre-exposure period to evaluate particle stability, followed by complete water replacement with freshly dosed solutions to maintain target concentrations. Throughout the experiment, no water changes were performed.

Precise environmental controls were implemented during the 10-day study period. water temperature was maintained at 18.5 ± 0.5 °C (hourly monitored), while a 14:10 light: dark photoperiod (3000 lux) was implemented. Dissolved oxygen levels were carefully maintained above 8 mg/L through continuous aeration and verified through twice-daily measurements. To evaluate treatment effectiveness, comprehensive water sampling was conducted from all tanks at regular intervals. These samples were analyzed for three key parameters: (1) standard water quality indicators, (2) microbial population counts, and (3) silver/zinc ion concentrations. This multi-faceted approach allowed for complete assessment of nanoparticle performance and potential environmental impacts.

### 2.5. Water Quality Parameters

Water quality parameters were systematically evaluated over a 192-h period following treatment application. Measurements were conducted at four critical time points: 6 h (initial response), 48 h (short-term effects), 120 h (mid-term stability), and 192 h (long-term impact). For precise measurement, specialized instruments from Hanna Instruments (GmbH, Germany) were employed: temperature was recorded using an alcohol thermometer, pH levels were measured with a calibrated pH meter, conductivity was assessed via conductivity meter, and dissolved oxygen concentrations were determined using a dedicated oxygen meter. This rigorous monitoring protocol enabled thorough characterization of temporal changes in water quality throughout the experimental duration.

The concentration of total dissolved solids (TDS, mg/L) can be simply determined using the conductivity (EC, micro-Siemens/cm) measurement. The relationship between these two parameters can be expressed with the following equation:

(1) $$TDS\left(mg/L\right)=k\times EC\;(\mu S/cm)$$

In natural water and wastewater, the relationship between TDS and EC becomes more evident when the TDS/EC ratio is around 0.64. Consequently, the value of k is 0.640 [30,31].

We analyzed key water quality parameters using standardized methods. Specifically, we measured ammonia nitrogen (NH_4_-N), nitrite nitrogen (NO_2_-N), nitrate nitrogen (NO_3_-N), and chemical oxygen demand (COD). These four parameters were directly analyzed using a DR1900 Hach Lange spectrophotometer with Hach Lange LCK test kits (Hach Lange, GmbH, Düsseldorf, Germany), strictly following the manufacturer’s protocols. For total phosphorus (PO_4_-P) determination, samples underwent a 15 min digestion process in an LT200 dry heater (Hach Lange, GmbH, Düsseldorf, Germany) equipped with dual heating blocks (Figure S3). Following this digestion, the processed samples were then measured using the same DR1900 spectrophotometer (Hach Lange, GmbH, Düsseldorf, Germany) to ensure methodological consistency across all analyses.

### 2.6. Enumeration of Aerobic Plate Count (APC)

To ensure sample integrity, two 100 mL water samples were aseptically collected from each aquarium using sterile, impermeable containers, adhering to standardized protocols [32]. For microbial enumeration, the aerobic plate count method was employed according to ISO 6222 [33], using Nutrient Agar (NA, Oxoid, UK). Briefly, samples were serially diluted in peptone diluent, and 0.1 mL aliquots from appropriate dilutions were inoculated onto NA plates via the pour plate technique.

Following inoculation, plates were incubated at 22 ± 2 °C for 48 h, after which colonies were enumerated. Only plates containing 30–300 colonies were counted to ensure statistical reliability, and results were calculated as colony-forming units per milliliter (CFU/mL). For comparative analysis, data were converted to logarithmic values (log_10_ CFU/mL). Microbial viability was monitored over 240 h post-treatment, with assessments conducted at 12 time points (1, 6, 24, 48, 72, 96, 120, 144, 168, 192, 216, and 240 h) to capture dynamic changes. All analyses were performed in duplicate to ensure methodological reproducibility.

### 2.7. Silver and Zinc Concentrations

First, two 100 mL water samples were collected from each aquarium using sterile, impermeable, and acid-washed containers to prevent contamination. For silver analysis, we employed the LCK 354 test kit, while zinc concentrations were determined using both LCK 360 and LCS 360 kits (Hach Lange, GmbH, Germany), strictly adhering to the manufacturer’s protocols. All spectrophotometric measurements were performed using DR1900 Hach Lange instruments to ensure consistency.

To further validate our silver concentration results, we conducted parallel measurements with an HI-97737 silver photometer (Hanna Instruments, GmbH, Germany), as illustrated in Figure S2. This dual-method approach provided robust verification of our findings.

The temporal monitoring of metal concentrations spanned 240 h post-treatment, with critical measurements taken at three strategic time points: 6 h (initial release), 120 h (mid-term stability), and 240 h (long-term persistence). This sampling regimen allowed us to track the dynamic changes in both silver and zinc concentrations throughout the experimental period.

### 2.8. Statistical Analyses

The data on water quality parameters, aerobic plate counts, and silver and zinc concentrations are reported as the mean ± standard deviation (SD) of the mean (*n* = 3). The normality of residuals and homogeneity of variances were assumed using the Shapiro–Wilk and Levene’s tests. To investigate the effects of treatment and time (interaction effect), all parameters were analyzed using mixed ANOVA. Additionally, One-way ANOVA was conducted to evaluate the significance of treatment effects. Tukey’s post hoc test was applied to further assess the effect of the treatment. The effect sizes of treatment, time, and interaction were calculated using Omega squared [34]. Cohen [35] and Field [36] have provided benchmarks to define very small (ω^2^ ˂ 0.01), small (0.01 ˂= ω^2^ ˂ 0.06), medium (0.06 ˂= ω^2^ ˂ 0.14), and large (ω^2^ >= 0.14) effects.

(2) Omega squared (ω2)=(df effect×(MS effect−MS error))/(SS total+MS error)

df = degree of freedom; MS = mean squares; SS = sum of squares.

The level of significance was *p* < 0.05. Statistical analyses were performed using R software version 4.3.1 (R, Vienna, Austria), GraphPad Prism 8.2.0 (GraphPad Software, Boston, MA, USA), and Origin 2020 (OriginLab, Northampton, MA, USA).

## 3. Results

### 3.1. Characterization of Nanoparticles

**Figure 1:**
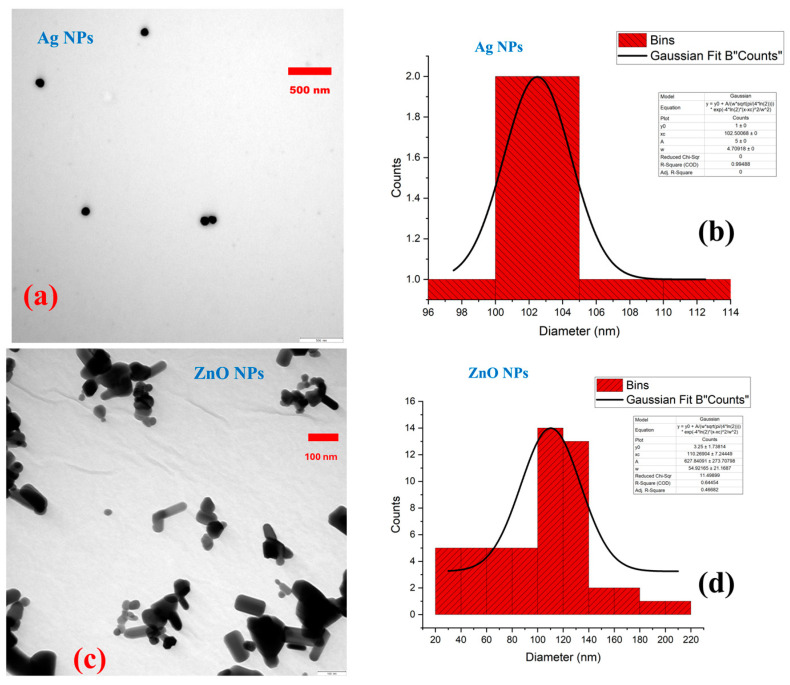
Characterization of silver nanoparticles (Ag NPs) and zinc oxide nanoparticles (ZnO NPs) with the aid of transmission electron microscopy (TEM). Ag NPs were spherical in shape and had an average particle size of 102.50 nm (scale bar: 500 nm) (**a**,**b**). ZnO NPs were cylindrical or rod-like structures and had an average particle size of 110.27 nm (scale bar: 100 nm) (**c**,**d**).

The morphology and size characteristics of the nanoparticles were determined using transmission electron microscopy (TEM) analysis (Figure 1). The silver nanoparticles (Ag NPs) exhibited a spherical shape with an average particle size of 102.50 ± 2.35 nm (Figure 1a,b). In contrast, the zinc oxide nanoparticles (ZnO NPs) were cylindrical or rod-like in structure and had an average size of 110.27 ± 27.46 nm (Figure 1c,d).

**Figure 2:**
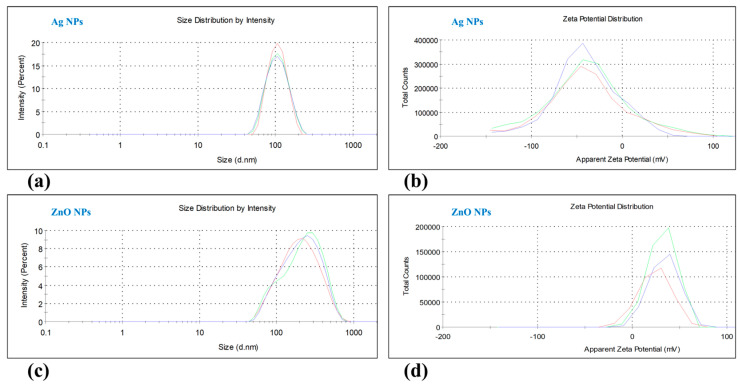
The particle size and zeta potential distribution of silver nanoparticles (Ag NPs) and zinc oxide nanoparticles (ZnO NPs) were analyzed using Dynamic Light Scattering (DLS); the particle size distribution of Ag NPs showed one peak at 100.5 nm (**a**). The zeta potential distribution of Ag NPs was −42.0 mv (**b**). The particle size distribution of ZnO NPs showed one peak at 212.3 nm (**c**). The zeta potential distribution of ZnO NPs was 31.7 mv (**d**).

The particle size distribution and zeta potentials of the nanoparticles were revealed using Dynamic Light Scattering (DLS) with a ZetaSizer NanoZS instrument (Figure 2). The Ag NPs showed a unimodal particle size distribution with a single peak at 100.5 nm and a zeta potential of −42.0 mV (Figure 2a,b). The ZnO NPs, on the other hand, exhibited a monomodal size distribution with a peak at 212.3 nm and a zeta potential of 31.7 mV (Figure 2c,d).

**Figure 3:**
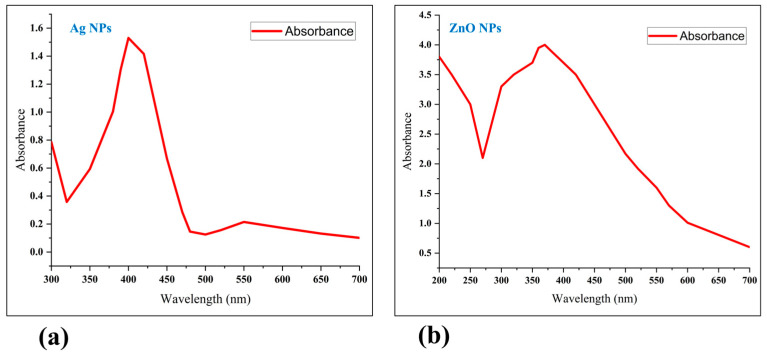
Characterization of silver nanoparticles (Ag NPs) and zinc oxide nanoparticles (ZnO NPs) using a UV–Vis spectrophotometer. The UV-Vis absorption spectrum of Ag NPs showed peak absorption (1.53) at 400 nm (**a**). The UV-Vis absorption spectrum of ZnO NPs showed peak absorption (4.0) at 370 nm (**b**).

The UV-Vis absorption spectra of the nanoparticles were also characterized (Figure 3). The Ag NPs displayed an absorption spectrum in the 300–700 nm range, with a peak absorption at 400 nm and a surface plasmon resonance (SPR) peak of 1.53 (Figure 3a). The ZnO NPs showed an absorption spectrum between 200 and 700 nm, with a peak at 370 nm and SPR peak of 4.0 (Figure 3b). The polydispersity index (PDI) values indicated that the Ag NPs were monodispersed (PDI = 0.073), while the ZnO NPs exhibited a polydispersed nature (PDI = 0.353).

### 3.2. Wastewater Characterization

The physical, chemical, and microbiological analyses of aquaculture wastewater are presented in Table 1. Ammonia nitrogen (NH_4_-N) and total phosphorus (PO_4_-P) concentrations were 2.34 mg/L and 1.66 mg/L, respectively, while the chemical oxygen demand (COD) and pH levels were 30.15 mg/L and 8.54. The aerobic plate count (APC) after 48 h of incubation at 22 °C was 5.562 Log_10_ CFU/mL. Silver and zinc concentrations were found to be 0.007 mg/L, and 0.14 mg/L.

### 3.3. Water Quality Parameters

**Figure 4:**
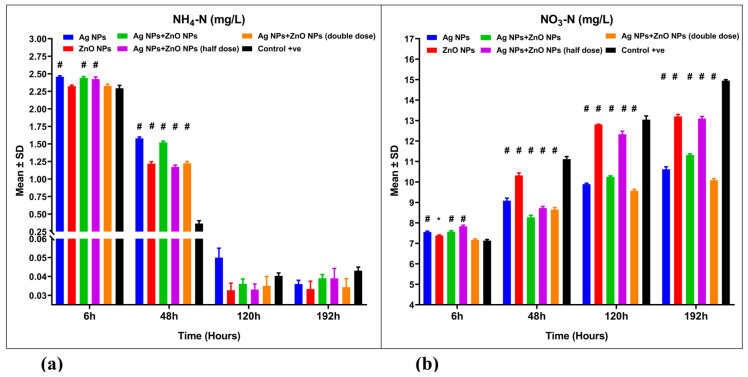
Water quality parameters for aquaculture wastewater treated with nanoparticles and untreated aquaculture wastewater (control + ve): (**a**) ammonia nitrogen (NH_4_-N, mg/L) and (**b**) nitrate nitrogen (NO_3_-N, mg/L). The bars display the mean ± SD of the mean (*n* = 3). Statistically significant differences were observed at *p* < 0.05 (ANOVA, Tukey’s post hoc). * (*p* < 0.05), and # (*p* < 0.001) when compared to the control group.

Ammonia nitrogen (NH_4_-N) levels in aquaculture wastewater treated with silver nanoparticles (Ag NPs) and zinc oxide nanoparticles (ZnO NPs), both singly and in combination, were assessed (Figure 4a). A mixed ANOVA revealed significant interaction effects [F(15, 48) = 358.4, *p* = 0.001, ω^2^ = 0.0285] (Table 2). The removal efficiencies of NH_4_-N in the nanoparticle-treated aquaculture wastewater were 98.46%, 98.58%, 98.33%, 98.33%, and 98.53%, respectively (Figure 4a, Table 3). These values are comparable to the control group (non-treated aquaculture wastewater), which had a removal efficiency of 98.16% (*p* > 0.05).

Nitrate nitrogen (NO_3_-N) levels showed significant differences in nanoparticle-treated aquaculture wastewater compared to the control group (*p* ˂ 0.001) (Figure 4b). A mixed ANOVA indicated significant differences in the interaction effect [F(15, 48) = 306.8, *p* = 0.001, ω^2^ = 0.1126] (Table 2).

**Figure 5:**
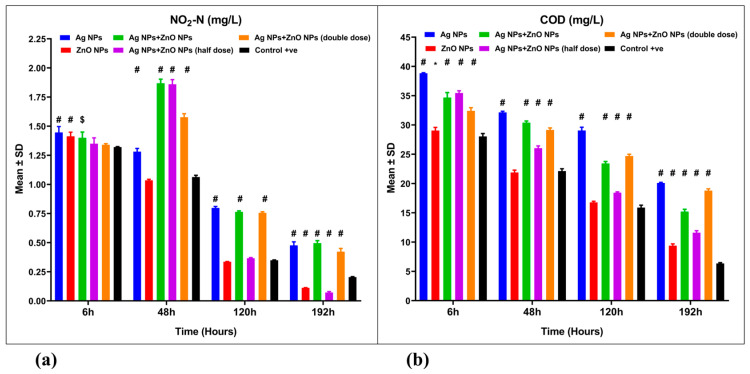
Water quality parameters for aquaculture wastewater treated with nanoparticles and untreated aquaculture wastewater (control + ve). (**a**) Nitrite nitrogen (NO_2_-N, mg/L) and (**b**) chemical oxygen demand (COD, mg/L). The bars display the mean ± SD of the mean (*n* = 3). Statistically significant differences were observed at *p* < 0.05 (ANOVA, Tukey’s post hoc). * (*p* < 0.05); $ (*p* < 0.01); and # (*p* < 0.001), when compared to the control group.

The removal efficiencies of nitrite nitrogen (NO_2_-N) in aquaculture wastewater treated with nanoparticles were 63.89%, 91.52%, 65.42%, 94.52%, and 67.98%, respectively (Figure 5a, Table 3). These values showed significant differences (*p* ˂ 0.001) when compared to the control group, which had a removal efficiency of 84.52%. The highest efficiency for NO_2_-N removal was observed in aquaculture wastewater treated with a combination of 0.025 mg Ag NPs and 0.5 mg ZnO NPs/L, reaching 94.52%. This was followed by the group treated with 1 mg ZnO NPs/L, which exhibited an efficiency of 91.52% (Table 3). A mixed ANOVA indicated significant differences in the interaction effect [F(15, 48) = 185.4, *p* = 0.001, ω^2^ = 0.225] (Table 2).

The removal efficiencies of chemical oxygen demand (COD) in aquaculture wastewater treated with nanoparticles were 33.33%, 68.82%, 49.59%, 61.49%, and 37.65%, respectively (Figure 5b, Table 3). These results showed significant differences (*p* ˂ 0.001) compared to the control group, which had a removal efficiency of 78.94%. The highest COD removal efficiency was found in untreated aquaculture wastewater (control group) at 78.94%. This was followed by the group treated with 1 mg ZnO NPs/L, which had an efficiency of 68.82% (Table 3). A mixed ANOVA revealed significant differences in the interaction effect [F(15, 48) = 57.55, *p* = 0.001, ω^2^ = 0.0256] (Table 2).

**Figure 6:**
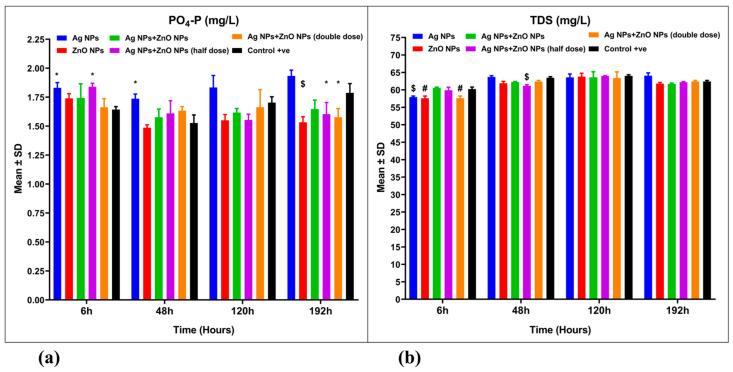
Water quality parameters for aquaculture wastewater treated with nanoparticles and untreated aquaculture wastewater (control + ve). (**a**) Total phosphorus (PO_4_-P, mg/L) and (**b**) total dissolved solids (TDS, mg/L). The bars display the mean ± SD of the mean (*n* = 3). Statistically significant differences were observed at *p* < 0.05 (ANOVA, Tukey’s post hoc). * (*p* < 0.05); $ (*p* < 0.01); and # (*p* < 0.001) when compared to the control group.

The removal efficiencies of total phosphorus (PO_4_-P) in aquaculture wastewater treated with 1 mg/L ZnO NPs, 0.025 mg/L Ag NPs combined with 0.5 mg/L ZnO NPs, and 0.1 mg/L Ag NPs combined with 2 mg/L ZnO NPs were measured at 7.68%, 3.41%, and 5.02%, respectively (Figure 6a, Table 3). These findings indicated significant differences (*p* < 0.001) when compared to the control group and the group treated with 0.05 mg Ag NPs/L, which negatively impacted total phosphorus removal. The highest removal efficiency of total phosphorus was observed in the group treated with 1 mg ZnO NPs/L, reaching 7.68% (Table 3). A mixed ANOVA revealed significant differences in the interaction effect [F(15, 48) = 3.549, *p* = 0.001, ω^2^ = 0.169] (Table 2).

Total dissolved solids (TDS) levels did not show significant differences between the nanoparticle-treated aquaculture wastewater groups and the control group (*p* > 0.05) (Figure 6b). Similarly, conductivity (EC) levels also did not exhibit significant differences when comparing nanoparticle-treated groups to the control group (Figure S4). However, a mixed ANOVA indicated significant differences in the interaction effect [F(15, 48) = 5.643, *p* = 0.001, ω^2^ = 0.111] (Table S1).

**Figure 7:**
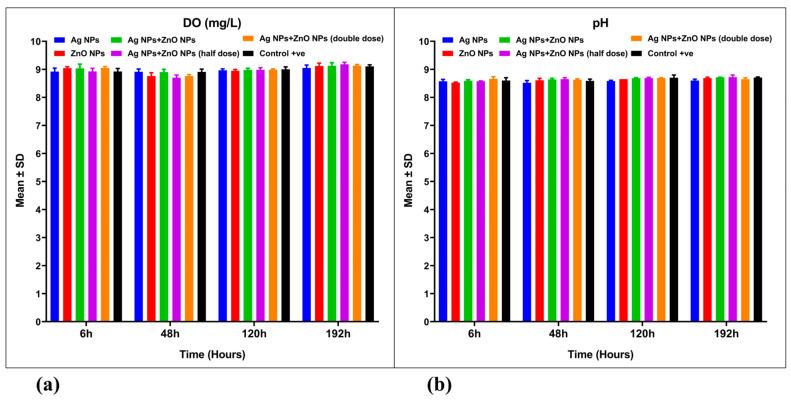
Water quality parameters for aquaculture wastewater treated with nanoparticles and untreated aquaculture wastewater (control + ve). (**a**) Dissolved Oxygen (DO, mg/L) and (**b**) pH. The bars represent the mean ± SD of the mean (*n* = 3). The bars display the mean ± SD of the mean (*n* = 3). Statistically significant differences were observed at *p* < 0.05 (ANOVA, Tukey’s post hoc).

Dissolved oxygen (DO) levels did not demonstrate significant differences between the nanoparticle-treated aquaculture wastewater groups and the control group (*p* > 0.05) (Figure 7a). A mixed ANOVA revealed no significant differences in the interaction effect [F(15, 48) = 1.651, *p* = 0.095, ω^2^ = 0.055] (Table 2). Likewise, pH levels also did not exhibit significant differences when comparing nanoparticle-treated groups to the control group (Figure 7a), with a mixed ANOVA showing no significant differences in the interaction effect [F(15, 48) = 1.183, *p* = 0.316, ω^2^ = 0.021] (Table 2). Throughout the experiment, both DO, and pH levels showed only minor fluctuations (Figure 7a,b).

### 3.4. Aerobic Plate Count (APC)

**Figure 8:**
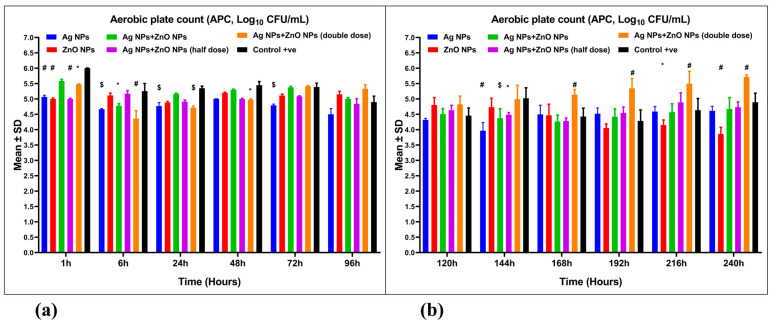
The aerobic plate count (APC, Log_10_ CFU/mL) of aquaculture wastewater treated with nanoparticles and untreated aquaculture wastewater (control + ve). (**a**) APC from 1 to 96 h and (**b**) APC from 120 to 240 h. The bars display the mean ± SD of the mean (*n* = 3). Statistically significant differences were observed at *p* < 0.05 (ANOVA, Tukey’s post hoc). * (*p* < 0.05); $ (*p* < 0.01); and # (*p* < 0.001) when compared to the control group.

Aerobic plate counts (APCs) were analyzed in aquaculture wastewater treated with Ag NPs and ZnO NPs, both singly and in combination (Figure 8a,b). Log reductions in APC for nanoparticle-treated wastewater were 1.191, 1.947, 1.133, 1.071, and 0.087, respectively, while the reduction in untreated wastewater was 0.911. The highest log reduction in APC was observed in the group treated with 1 mg ZnO NPs/L, with a reduction of 2 logs, followed by 0.05 mg Ag NPs/L with a 1.191 log reduction. Conversely, the combination treatment of 0.1 mg/L Ag NPs and 2 mg/L ZnO NPs showed the lowest APC reduction, with less than 0.1 log. The 1 mg ZnO NPs/L showed significant differences (*p* ˂ 0.001) compared to the control and the 0.05 mg Ag NPs/L group (Figure 8a,b). A mixed ANOVA revealed a significant interaction effect [F(55, 144) = 7.689, *p* = 0.001, ω^2^ = 0.323] (Table 4).

The group treated with 0.05 mg Ag NPs/L showed the highest APC reduction of 1.839 log at 144 h post-treatment, while the 0.1 mg/L Ag NPs combined with 2 mg/L ZnO NPs group had a maximum reduction of 1.444 log at 6 h post-treatment (Figure 8a,b). At 168 h, groups treated with 0.05 mg/L Ag NPs + 1 mg/L ZnO NPs and 0.025 mg/L Ag NPs + 0.5 mg/L ZnO NPs had the highest APC reductions of 1.544 and 1.520 log, respectively. In the control group, untreated wastewater reached a 1.521 log reduction at 192 h post-treatment (Figure 8b).

### 3.5. Silver and Zinc Concentrations

**Figure 9:**
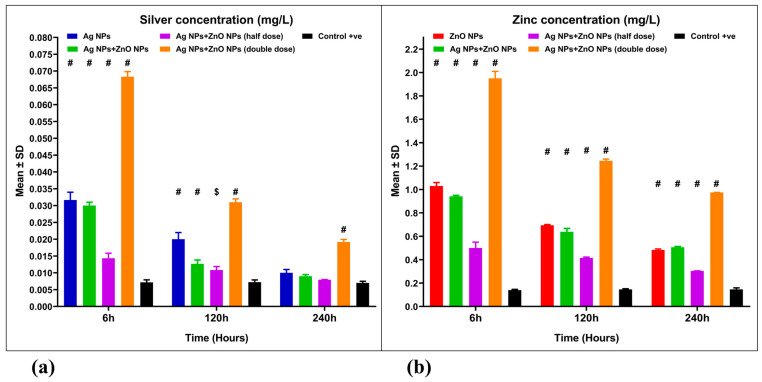
Silver and Zinc concentrations for aquaculture wastewater treated with nanoparticles and untreated aquaculture wastewater (control + ve): (**a**) Silver concentration (Ag, mg/L) and (**b**) Zinc concentration (Zn, mg/L). The bars display the mean ± SD of the mean (*n* = 3). Statistically significant differences were observed at *p* < 0.05 (ANOVA, Tukey’s post hoc). $ (*p* < 0.01); and # (*p* < 0.001), when compared to the control group.

Silver (Ag) and zinc (Zn) concentrations were measured in aquaculture wastewater treated with Ag NPs and ZnO NPs, both singly and in combination (Figure 9a,b). Silver concentrations in wastewater that treated with 0.05 mg Ag NPs/L, 0.05 mg/L Ag NPs + 1 mg/L ZnO NPs, and 0.025 mg/L Ag NPs + 0.5 mg/L ZnO NPs were 0.01, 0.009, and 0.0079 mg/L, respectively (Figure 9a), with no significant differences (*p* > 0.05) from the control group (0.007 mg/L). In contrast, the 0.1 mg/L Ag NPs + 2 mg/L ZnO NPs treatment revealed significant differences (*p* ˂ 0.001) compared to the control (Figure 9a). A mixed ANOVA revealed a significant interaction effect [F(8, 30) = 208.9, *p* = 0.001, ω² = 0.214] (Table 5).

Zinc concentration in wastewater treated with 1 mg ZnO NPs/L, 0.05 mg/L Ag NPs + 1 mg/L ZnO NPs, 0.025 mg/L Ag NPs + 0.5 mg/L ZnO NPs and 0.1 mg/L Ag NPs + 2 mg/L ZnO NPs were 0.483, 0.506, 0.304, and 0.974 mg/L, respectively (Figure 9b), with significant differences (*p* ˂ 0.001) compared to the control group (0.147 mg/L). A mixed ANOVA showed a significant interaction effect [F(8, 30) = 196,0, *p* = 0.001, ω² = 0.0884] (Table 5).

## 4. Discussion

The UV–Vis absorbance spectrum displayed a characteristic peak for zinc oxide nanoparticles (ZnO NPs) at 370 nm, which is attributed to the phenomenon of surface plasmon resonance (SPR) in these plasmonic ZnO NPs [37]. The wavelength range of 350–380 nm has been shown to include the greatest absorption of ZnO NPs, which is a typical absorption range for ZnO NPs [38]. TEM is critical for detailed visualizations of Ag and ZnO nanoparticles morphology, size, and shape, which are crucial for understanding their physical properties and potential applications in wastewater treatment [17,20,28]. The size estimation of zinc oxide nanoparticles (ZnO NPs) and silver nanoparticles (Ag NPs) obtained through Dynamic Light Scattering (DLS) is slightly larger than the sizes measured from transmission electron microscopy (TEM) images. This discrepancy arises from the different measurement techniques: DLS accounts for the solvent hydration shell surrounding the particles, while TEM images are taken of dried samples. Similar findings have been reported in other studies [38,39]. Silver nanoparticles (Ag NPs) give a strong surface plasmon band in the visible region, centered at wavelengths between 395 and 425 nm, as previously reported [40,41]. The lowest polydispersity index (PDI) of silver nanoparticles suggests that aggregates were more uniform in size than zinc oxide nanoparticles. ZnO NPs are not uniform in size; they range from 20 to 200 nm, exhibiting a high polydispersity index (PDI). This indicates the presence of both larger and smaller aggregates [38,42]. ZnO NPs tend to self-aggregate, which increases particle size and reduces their biocidal efficiency [38].

Nitrification is an oxidative process that transforms reduced forms of inorganic and organic nitrogen, primarily ammonia, into nitrate. The process is mediated by microorganisms and contributes to the movement of nitrogen through the biogeochemical nitrogen cycle, which occurs in two steps. In the first step, ammonia-oxidizing bacteria (AOB) oxidize ammonia (NH_4_^+^) to nitrite (NO_2_^−^) and in the second step, nitrite (NO_2_^−^) is oxidized to nitrate (NO_3_^−^) by the nitrite-oxidizing bacteria (NOB) [43,44].

1. NH_4_^+^ + CO_2_ + 1.5 O_2_ + *Nitrosomonas* → NO_2_^−^ + H_2_O + H^+^ (first step)
2. NO_2_^−^ + 0.5 O_2_ + *Nitrobacter* → NO_3_^−^ (second step)

Biological removal of ammonia is accomplished by a sequential nitrification process, facilitated by ammonium-oxidizing bacteria (AOB) and nitrite-oxidizing bacteria (NOB). According to Yang et al. [45], nitrifiers are particularly sensitive to stressors, such as nanoparticles (NPs). Klotz and Stein [46] suggested that nitrifiers are highly sensitive to chemical stressors due to their limited energy harvesting capabilities linked to their chemolithoautotrophic metabolism, or due to physiological limitations that affect their resilience. Marcilhac et al. [47] noted that the sensitivity of NOB means that any reduction in their growth can completely halt the conversion of NO_2_-N to NO_3_-N, which may explain the decreased performance of reactors when treated with 50 mg NPs/L [48].

In the sequencing batch reactors (SBR) dosed with 1 mg ZnO NPs/L, the removal efficiencies of total nitrogen (TN) and COD were 71.6%, and 95.7%, respectively. In the control reactors, these efficiencies were 71.1% for TN and 96.1% for COD. Notably, the average COD levels in treated reactors were even lower than those in the control [48,49]. These findings suggest that dosing of Ag NPs, ZnO NPs or their combinations did not significantly alter COD removal efficiencies. The average removal efficiencies in reactors treated with Ag-NPs (up to 0.5 mg/L) were comparable to those of the control reactors [49]. Overall, these results indicate that Ag NPs, alone or combined with ZnO NPs, do not significantly affect COD and NH_4_-N removal efficiency in wastewater treatment.

High concentrations of NPs and Zn^2^^+^ can inhibit both nitrifiers and denitrifiers [48]. Additionally, Ag NPs at concentration of 0.5 mg/L were found to slightly inhibit the respiration of nitrifying bacteria [49]. Several studies have indicated that exposure to 1 mg/L of Ag NPs significantly inhibits nitrification [50,51]. This inhibition could impede biological nitrogen removal in wastewater treatment plants (WWTPs) [50].

In the current study, wastewater treatment with Ag NPs (0.05–0.1 mg/L) combined with ZnO NPs (0.5–2 mg/L) resulted in a slight inhibition of NOB, as evidenced by an increase in NO_2_-N levels 48 h post-treatment compared to the control. The removal efficiency of NO_2_-N in wastewater treated with either 1 mg/L ZnO NPs or 0.5 mg/L ZnO NPs combined with 0.025 mg/L Ag NPs showed the highest removal efficiency (91.52% and 94.52%) compared to control (84.52%). These results are likely attributed to the recovery of the respiration of nitrifying microorganisms. Similar findings have been reported in previous studies [48,49]. Yang et al. [45] reported that when nitrifiers were exposed to a low concentration of sublethal silver nanoparticles (0.025 mg/L), there was no significant impact on nitrogen cycling. However, at higher concentrations ranging from 0.05 to 0.5 mg/L, the potential for particle aggregation increases [27,47]. This aggregation can reduce the available surface area for reactions, thereby diminishing overall removal efficiency. Consequently, nitrogen cycling is significantly affected, leading to a lesser inhibition of nitrite nitrogen removal when compared to zinc oxide nanoparticles (ZnO NPs) used alone or in combination with a lower concentration of Ag NPs (0.025 mg/L). Furthermore, at elevated concentrations, the interaction between the two types of nanoparticles may impede overall efficiency when used together, due to factors such as competition for active sites or alterations in particle aggregation [28,29,48,49,50,51].

Zinc oxide nanoparticles (ZnO NPs) play a vital role in the degradation of pollutants in wastewater. When these nanoparticles are exposed to light, they generate electron–hole pairs. These pairs then interact with oxygen and water, resulting in the formation of highly reactive species, such as hydroxyl radicals [52]. The low concentrations of reactive oxygen species (ROS) produced by ZnO NPs can stimulate microorganisms to secrete polysaccharides and proteases, thereby enhancing the performance of activated sludge. This process ultimately promotes the removal of ammonia nitrogen and nitrite nitrogen from sewage [53]. However, it is important to note that excessive ROS generation may be a primary mechanism behind the toxicity of nanoparticles. High levels of ROS can induce oxidative DNA damage, protein denaturation, and lipid peroxidation [54]. Thus, while ZnO NPs can be beneficial in wastewater treatment, careful consideration must be given to the balance of ROS production to mitigate potential toxic effects.

The inhibition of anammox activity by silver nanoparticles (Ag NPs) is primarily due to the release of Ag^+^ ions. These Ag^+^ ions can react with organic matter (OM), reducing its availability for microbial utilization, which has been shown to negatively impact anammox activity [55,56]. Moreover, Ag^+^ can increase the redox potential (Eh), which further inhibits anammox activity by limiting the electron donation from ferrous iron and organic compounds [57]. In addition to these effects, the toxicity of Ag NPs can lead to a reduction in extracellular polymeric substances (EPS). This reduction compromises the protective capabilities of anammox granules and increases the production of reactive oxygen species (ROS), ultimately resulting in decreased anammox activity [53]. Thus, the interplay of these factors highlights the detrimental impact of Ag NPs on anammox processes in wastewater treatment.

The initial nitrate nitrogen (NO_3_-N) concentration in wastewater was 6.99 mg/L, which increased to more than 10 mg/L in samples treated with nanoparticles, while the control group reached 15 mg/L. Nanoparticle-treated wastewater and control groups experienced nitrate accumulation due to nitrification, with potential concentrations exceeding 25 mg NO_3_-N/L in surface water and 100 mg NO_3_-N/L in ground water [58]. The control group had significantly higher NO_3_-N levels than the nanoparticle-treated wastewater, particularly with regard to the lowest concentration, found in samples treated with 0.1 mg/L Ag NPs combined with 2.0 mg/L ZnO NPs. Denitrification, a key biological process in wastewater treatment that occurs under anaerobic or anoxic conditions, can be influenced by Ag NPs [59]. This process involves nitrate reductase (NR) and nitrite reductase (NIR) [60]. High concentrations of ZnO NPs and Ag NPs decreased the activity of NR but had little effect on NIR activity [59,61]. Our findings are consistent with the observation of higher effluent NO_3_-N concentrations compared to initial levels [61].

In the present study, the removal efficiency of total phosphorus (PO_4_-P) in wastewater treated with 1 mg/L ZnO NPs was 7.68%. Notably, treatment with 0.05 mg/L of Ag NPs resulted in no PO_4_-P removal when compared to the control. The removal efficiency observed, particularly with 1 mg/L ZnO NPs, is influenced by the adsorption properties of ZnO NPs. ZnO NPs have a positive zeta potential, suggesting that they may adsorb negatively charged phosphorus ions (such as PO_4_^3-^) in wastewater. This potential for adsorption likely contributes to the observed decrease in total phosphorus levels, especially when compared to the control group and Ag NPs. Zheng et al. [61] reported similar results, indicating that 1 mg/L of ZnO NPs had no significant impact on phosphorus removal. In contrast, Daraei et al. [48] found that phosphorus removal efficiency improved with exposure to 10 and 50 mg ZnO NPs/L. They noted that higher concentrations of ZnO NPs inhibited phosphorus removal due to the release of zinc ions from the NPs and increased production of reactive oxygen species (ROS), which adversely affected polyphosphate accumulating organisms (PAOs) and polyphosphate kinase (PPK) activity [48,61]. Specifically, 50 mg/L of ZnO NPs significantly inhibited PPK activity, which is important for phosphorus removal under aerobic conditions, while lower concentrations did not have a notable effect on PPK activity [48]. The slight phosphorus removal observed at low doses of ZnO NPs in this study was attributed to the requirement of certain enzymes for co-factors, as metal ions are essential for substrate binding to enzyme molecules [62,63].

Dissolved oxygen (DO) concentration is a crucial factor in biological nutrient removal (BNR) processes and significantly affect operational costs [64]. Most WWTPs maintain DO levels above 2 mg/L during the aerobic stage of BNR to ensure complete nitrification of ammonia to nitrate and to establish stable populations of AOB and NOB [65]. The high removal efficiencies of ammonium nitrogen (NH_4_-N) and nitrite nitrogen (NO_2_-N) observed in the control group can be attributed to elevated dissolved oxygen (DO) levels, which are above 8 mg/L. Specifically, adequate DO enhances the activity of aerobic bacteria, such as ammonia-oxidizing bacteria (AOB) and nitrite-oxidizing bacteria (NOB). These microorganisms are essential for breaking down organic matter and pollutants in wastewater. Moreover, they require oxygen to effectively decompose waste, which in turn reduces toxic substances and improves overall water quality. Consequently, high DO levels help mitigate the accumulation of NH_4_-N and NO_2_-N by facilitating the oxidation of these compounds, thereby decreasing their concentrations in the water [27,64,65].

In the present study, wastewater treated with a combination of 0.1 mg/L Ag NPs and 2 mg/L ZnO NPs exhibited a peak APC reduction of 1.444 log after 6 h. In contrast, the greatest reduction, of 1.839 log, occurred with 0.05 mg/L Ag NPs at 144 h. Furthermore, at 168 h, the treatments of 0.05 mg/L Ag NPs with 1 mg/L ZnO NPs and 0.025 mg/L Ag NPs with 0.5 mg/L ZnO NPs resulted in reductions of 1.544 and 1.520 log, respectively. Meanwhile, the control group attained a reduction of 1.521 log at 192 h. Lastly, the group treated with 1 mg/L ZnO NPs showed approximately a 2-log reduction at 240 h post-treatment.

The antimicrobial efficiency of metallic nanoparticles (MNPs) can be influenced by several factors, including their size, shape, concentration, contact time, pathogen load, pathogen species, water quality and the presence of UV irradiation. These variables play a significant role in determining the mechanisms underlying their antimicrobial activity [66]. Specifically, the duration of exposure to Ag NPs and their concentration were found to significantly affect bacterial production [66]. A complete inhibition of bacterial production occurred after just 1 h of exposure to Ag NPs across all tested concentrations (ranging from 0.05 to 10 mg/L). Furthermore, after a 48-h exposure period, bacterial production showed signs of recovery at lower concentrations of Ag NPs (0.05 and 0.1 mg/L), whereas it remained suppressed at higher concentrations (1 and 10 mg/L) [66].

The removal percentage of bacteria was found to increase with longer contact time and higher concentrations of nanoparticles. Notably, Ag NPs have demonstrated superior disinfection efficacy compared to ZnO NPs [17]. Furthermore, the use of combination of Ag NPs and ZnO NPs has proven to be an effective approach for eliminating indicator bacteria from contaminated water [17,67]. Specifically, Ag NPs at a concentration of 0.1 mg/L, when combined with ZnO NPs at 2 mg/L, showed the highest bacterial removal rate [67]. Similarly, Venis and Basu [17] found that a combination of 0.67 mg/L Ag NPs and 0.33 mg/L of ZnO NPs provided enhanced disinfection over a 5 h period compared to the use of ZnO NPs alone at a concentration of 1 mg/L.

Extensive research has demonstrated silver nanoparticles’ remarkable antimicrobial efficacy, with proven effectiveness against over 700 microbial species commonly found in wastewater treatment systems. These nanoparticles employ multiple bactericidal mechanisms that collectively target microorganisms. Most notably, they compromise cellular integrity by disrupting membrane structure, impair respiratory functions, and induce severe oxidative stress [68]. The primary antimicrobial action stems from the sustained release of biologically active Ag^+^ ions from the nanoparticle surface. Through various surface interactions, these ionic silver species inflict substantial damage to microbial cells [17]. The released Ag^+^ ions exhibit strong affinity for negatively charged components of bacterial cell walls. This interaction triggers a cascade of detrimental effects: critical cellular enzymes become deactivated, while membrane permeability becomes dysregulated [18,19]. The cumulative damage ultimately results in complete cell lysis and microbial death. Furthermore, the antimicrobial potency extends to genetic material, as Ag^+^ ions readily bind to microbial DNA. This binding interferes with essential cellular transport systems, particularly those governing salt and phosphorus uptake [19,69]. Such comprehensive interference with fundamental metabolic processes explains silver nanoparticles’ exceptional effectiveness in microbial inactivation across diverse wastewater treatment applications.

In contrast to Ag NPs, zinc oxide nanoparticles (ZnO NPs) trigger a pronounced oxidative stress response in bacteria cells by generating excessive intracellular reactive oxygen species (ROS). This distinctive property stems primarily from ZnO NPs’ unique ability to produce ROS when exposed to UV radiation or visible light [70]. The resulting oxidative stress causes significant cellular damage through multiple mechanisms: ROS induce lipid peroxidation that compromises bacterial membrane integrity, ultimately leading to apoptotic cell death. Furthermore, the gradual release of zinc ions from the nanoparticles disrupts critical metabolic processes and inhibits essential enzyme activities, creating an additional bactericidal effect [20]. The antimicrobial action of ZnO NPs is enhanced by their surface charge characteristics in aqueous environments. Typically developing a positive charge due to surface defects or specific functional groups, ZnO NPs readily adhere to negatively charged bacterial membranes through electrostatic attraction [70]. This targeted interaction increases local ROS concentration at the cell surface, amplifying the oxidative damage. Extensive research has validated the remarkable disinfection capabilities of both silver and zinc oxide nanoparticles in water treatment applications. Multiple studies confirm that even at minimal concentrations, these nanoparticles achieve significant microbial population reductions [17,23,25,39,71], demonstrating their potential as effective water purification agents.

Dissolution, aggregation, and agglomeration are the main factors affecting the state of metal NPs in suspensions. Various environmental factors, such as ionic strength, pH, dissolved oxygen, and natural organic matter (NOM), can further influence the dissolution and aggregation of metal NPs [27,72]. All these factors will consequently impact the bioavailability, uptake, and toxicity of NPs. Specifically, the release of Zn^2^^+^ from ZnO NPs in wastewater is more pronounced in acidic conditions and low ionic strength. In contrast, under alkaline conditions, ZnO NPs tend to adhere strongly to sewage sludge rather than remaining dissolved or dispersed in the filtrate [61,73].

The zeta potential serves as a critical indicator of colloidal interactions, particularly between nanoparticles (NPs) and natural organic matter (NOM). This parameter reflects the electrostatic repulsion between particles and is highly influenced by solution chemistry, such as pH and ionic strength [74]. In this context, Ag NPs typically exhibit a negative zeta potential, whereas ZnO NPs carry a positive charge. This opposing charge suggests that, under certain conditions, electrostatic attraction could dominate, leading to aggregation. However, stability can be maintained through steric hindrance (e.g., from capping agents) or unfavorable environmental factors (e.g., extreme pH or high NOM concentrations) [75].

In our study, citrate-coated Ag NPs were used, which are electrostatically stabilized. Due to the enhanced negative charge from citrate, steric hindrance likely reduces aggregation even when mixed with oppositely charged ZnO NPs [76]. Similarly, NOM (e.g., humic/fulvic acids) adsorbs onto NPs, forming steric or electrostatic barriers that further inhibit aggregation [75]. By contrast, studies such as Jiang et al. [77] observed rapid heteroaggregation between oppositely charged NPs in aqueous suspensions, while Wang et al. [74] demonstrated aggregation in low-NOM or pure water conditions.

Further evidence comes from Dutta et al. [78], who reported that mixing Ag NPs with ZnO NPs reduced the zeta potential of the colloidal suspension over time. This phenomenon likely arises from aggregation driven by ion adsorption, electrostatic interactions, or other factors, ultimately altering the NPs’ surface charge. As a result, decreased zeta potential weakens interparticle repulsion, leading to aggregation and an increase in hydrodynamic size [78]. Consistently, Wang et al. [74] documented heteroaggregation between Ag and ZnO NPs in freshwater, forming larger clusters. As a result, the Ag NPs and ZnO NPs aggregates in mixed systems. Consequently, these aggregates exhibit lower efficiency in removing contaminants (e.g., NO_2_^−^N and COD) and inactivating microbes compared to ZnO NPs alone.

Zinc oxide nanoparticles (ZnO NPs) and silver nanoparticles (Ag NPs) are commonly utilized in aquaculture due to their distinctive antimicrobial application, biocidal properties, and cytotoxicity [29]. Both silver and zinc oxide NPs are regarded as nanomediators, which exhibit relatively low toxicity and minimal adverse effects when used in limited quantities. However, excessive use can pose hazard to both organisms and the environment [79]. According to European union reports, the concentrations of Ag NPs in surface water ranged from 0.06 to 16 ng/L, while ZnO NPs ranged from 1.7 to 21 μg/L [80]. The Environmental Protection Agency (EPA) has established that drinking water should not exceed 5 mg of zinc per liter. Furthermore, the World Health Organization (WHO) states that silver concentrations in natural waters typically range from 0.2 to 0.3 mg/L. In standard drinking water, Ag is between “nondetectable” and 0.1 mg/L [81].

In the present study, the concentrations of silver and zinc in aquaculture wastewater (control samples) were measured at 0.007 mg/L, and 0.147 mg/L, respectively. In contrast, the wastewater treated with nanoparticles showed silver concentrations between 0.0079 and 0.01 mg/L, while zinc levels ranged from 0.304 to 0.974 mg/L. These results were consistent with the acceptable limits set by WHO and EPA.

## 5. Conclusions

The study highlights the potential of zinc oxide nanoparticles (ZnO NPs) and silver nanoparticles (Ag NPs) as effective and sustainable solutions for treating aquaculture wastewater. Specifically, the application of 1 mg/L ZnO NPs led to significant reductions in NH_4_-N, NO_2_-N, and COD by 98.58%, 91.52%, and 68.82%, respectively. Furthermore, the treated wastewater showed a 2-log reduction in microbial populations. In contrast, Ag NPs (0.05 mg/L) showed limited efficacy, with lower NO_2_-N (63.89%), and COD (33.33%) removal and 1.8 log reduction in microbial populations. Notably, mixed Ag-ZnO NP treatments displayed reduced efficiency compared to ZnO NPs alone, likely due to aggregation-induced particle deactivation, which hindered their reactivity.

While the control group demonstrated measurable pollutant removal through 10-day vigorous aeration and maintained dissolved oxygen levels above 8 mg/L, its performance was consistently surpassed by ZnO NPs treatments. Specifically, the untreated system achieved only 84.52% NO_2_-N removal and showed limited microbial reduction—significantly lower than ZnO NP-treated samples. This performance gap clearly establishes that nanoparticle-enhanced treatment offers substantial advantages over conventional aeration methods.

Importantly, our analysis revealed that residual silver and zinc concentrations in the treated wastewater consistently remained below the maximum permissible limits established by environmental regulatory agencies. This critical finding demonstrates that ZnO NP treatment can be implemented while maintaining compliance with environmental safety standards. These results collectively highlight three key advantages of ZnO nanoparticle wastewater treatment: achieving superior treatment efficiency at relatively low concentrations, minimal residual metal discharge within regulatory limits, and consistent performance across multiple pollutant categories.

To advance nanoparticle water treatment technology, future research should prioritize three interconnected objectives: (1) enhancing mixed NP stability through surface engineering and tailored capping agents to maximize Ag-ZnO synergies while preventing aggregation; (2) refining synthesis protocols via green chemistry, morphological control, and strategic doping to boost catalytic performance; and (3) developing practical NP recovery systems (e.g., magnetic separation, membrane filtration) to facilitate reuse and prevent environmental discharge. Concurrently, comprehensive life-cycle analyses and pilot-scale trials across varied wastewater streams will be essential to validate ecological safety and operational viability. These integrated efforts will bridge the gap between laboratory innovation and scalable, sustainable implementation, ultimately realizing the full potential of nanoremediation for water purification.

## Figures and Tables

**Table 1:** Physical, chemical, and microbiological analyses of aquaculture wastewater.

Parameters	Measurement
Temperature	18 °C
pH	8.54
Conductivity	94.1 μs/cm *
Total dissolved solids (TDS)	60.22 mg/L
Ammonia nitrogen (NH_4_-N)	2.34 mg/L
Nitrite nitrogen (NO_2_-N)	1.32 mg/L
Nitrate nitrogen (NO_3_-N)	6.99 mg/L
Chemical oxygen demand (COD)	30.15 mg/L
Total phosphorus (PO_4_-P)	1.66 mg/L
Silver (Ag)	0.007 mg/L
Zinc (Zn)	0.14 mg/L
Aerobic plate count (APC)	5.562 Log_10_ CFU/mL

* μs/cm: MicroSiemens per centimeter.

**Table 2:** ANOVA summary table for water quality parameters.

	Source of Variation	*df*	SS	MS	*F*	*p*	Effect Size (ω^2^)
Ammonia nitrogen (NH_4_-N, mg/L)	Treatment	5	0.8988	0.1798	481.2	0.001	0.0127
	Time	3	67.46	22.49	60,192	0.001	0.958
	Interaction	15	2.008	0.1339	358.4	0.001	0.0285
	Residual (Error)	48	0.0179	0.00037			
	Total	71	70.39				
Nitrate nitrogen (NO_3_-N, mg/L)	Treatment	5	68.66	13.73	1586	0.001	0.1944
	Time	3	243.9	81.29	9390	0.001	0.6912
	Interaction	15	39.84	2.656	306.8	0.001	0.1126
	Residual (Error)	48	0.4155	0.008657			
	Total	71	352.8				
Nitrite nitrogen (NO_2_-N, mg/L)	Treatment	5	1.623	0.1262	476.8	0.001	0.029
	Time	3	18.08	6.028	8854	0.001	0.836
	Interaction	15	1.893	0.3246	185.4	0.001	0.225
	Residual (Error)	48	0.03268	0.000681			
	Total	71	21.63				
Chemical oxygen demand (COD, mg/L)	Treatment	5	1237	247.4	1610	0.001	0.2427
	Time	3	3717	1239	8062	0.001	0.7297
	Interaction	15	132.7	8.844	57.55	0.001	0.0256
	Residual (Error)	48	7.376	0.1537			
	Total	71	5093				
Total phosphorus (PO_4_-P, mg/L)	Treatment	5	0.4492	0.08985	17.00	0.001	0.354
	Time	3	0.2045	0.06818	12.90	0.001	0.158
	Interaction	15	0.2813	0.01876	3.549	0.001	0.169
	Residual (Error)	48	0.2537	0.005285			
	Total	71	1.189				
Total dissolved solids (TDS, mg/L)	Treatment	5	14.28	2.855	5.897	0.001	0.039
	Time	3	225.0	75.02	154.9	0.001	0.736
	Interaction	15	40.98	2.732	5.643	0.001	0.111
	Residual (Error)	48	23.24	0.4842			
	Total	71	303.5				
Dissolved Oxygen (DO, mg/L)	Treatment	5	0.0275	0.0055	0.6824	0.682	0.009
	Time	3	0.7909	0.2636	32.68	0.001	0.542
	Interaction	15	0.1998	0.0133	1.651	0.095	0.055
	Residual (Error)	48	0.3872	0.0081			
	Total	71	1.405				
pH	Treatment	5	0.07633	0.01527	5.359	0.001	0.166
	Time	3	0.1067	0.03558	12.49	0.001	0.263
	Interaction	15	0.05056	0.003370	1.183	0.316	0.021
	Residual (Error)	48	0.1367	0.002849			
	Total	71	0.3704				

Note: df = degree of freedom; SS = sum of squares; MS  =  mean squares; (ω^2^) = omega squared.

**Table 3:** Removal efficiencies of ammonia nitrogen (NH_4_-N), nitrite nitrogen (NO_2_-N), total phosphorus (PO_4_-P), and chemical oxygen demand (COD) from aquaculture wastewater.

Water Parameters	Ag NPs (0.05 mg/L)	ZnO NPs (1 mg/L)	Ag NPs (0.05 mg/L) + ZnO NPs (1 mg/L)	Ag NPs (0.025 mg/L) + ZnO NPs (0.5 mg/L)	Ag NPs (0.1 mg/L) + ZnO NPs (2 mg/L)	Control + ve (Untreated)
NH_4_-N (mg/L)	98.46%	98.58%	98.33%	98.33%	98.53%	98.16%
NO_2_-N (mg/L)	63.89%	91.52%	65.42%	94.52%	67.98%	84.52%
COD (mg/L)	33.33%	68.82%	49.59%	61.49%	37.65%	78.94%
PO_4_-P (mg/L)	−16.47%	7.68%	0.80%	3.41%	5.02%	−7.63%

Negative (−) values indicate that there is no removal of PO_4_-P; instead, PO_4_-P tends to accumulate.

**Table 4:** ANOVA summary table for aerobic plate count (APC, Log_10_ CFU/mL).

Source of Variation	*df*	SS	MS	*F*	*p*	Effect Size (ω^2^)
Treatment	5	6.999	1.400	35.46	0.001	0.151
Time	11	15.59	1.417	35.90	0.001	0.337
Interaction	55	16.70	0.3036	7.689	0.001	0.323
Residual (Error)	144	5.685	0.03948			
Total	215	44.97				

Note: df = degree of freedom; SS = sum of squares; MS  =  mean squares; (ω^2^) = omega squared.

**Table 5:** ANOVA summary table for Zinc and silver concentrations.

	Source of Variation	*df*	SS	MS	*F*	*p*	Effect Size (ω^2^)
Silver Concentration (Ag, mg/L)	Treatment	4	0.00567	0.00142	986.5	0.001	0.507
	Time	2	0.00307	0.00154	1069	0.001	0.275
	Interaction	8	0.00240	0.0003	208.9	0.001	0.214
	Residual (Error)	30	0.00004	0.0000014			
	Total	44	0.01118				
Zinc Concentration (Zn, mg/L)	Treatment	4	7.822	1.955	3392	0.001	0.7687
	Time	2	1.431	0.7157	1242	0.001	0.1406
	Interaction	8	0.9038	0.1130	196.0	0.001	0.0884
	Residual (Error)	30	0.01729	0.00058			
	Total	44	10.17				

Note: df = degree of freedom; SS = sum of squares; MS  =  mean squares; (ω^2^) = omega squared.

## Data Availability

All data can be found in the manuscript and its Supplementary Files.

## Supplementary Materials

The following supporting information can be downloaded at https://www.mdpi.com/article/10.3390/nano15070559/s1: Figure S1: Water quality kits (All these kits were obtained from Hach Lange, GmbH, Germany); Figure S2: Silver and zinc kits (All these kits were obtained from Hach Lange, GmbH, Germany). HI-93737-01 reagents and HI-97737 Silver Photometer (Hanna Instruments, GmbH, Germany); Figure S3: DR1900 Hach Lange spectrophotometer and LT200 dry heater equipped with two blocks (Hach Lange, GmbH, Germany); Figure S4: The conductivity (EC, μS/cm) of aquaculture wastewater treated with nanoparticles was compared to that of untreated aquaculture wastewater (control + ve). The bars display the mean ± SD of the mean (n = 3). Statistically significant differences were observed at p < 0.05 (ANOVA, Tukey’s post hoc). * (*p* < 0.05), $ (*p* < 0.01), and # (*p* < 0.001) when compared to the control group; Table S1: ANOVA summary table for conductivity (EC, μS/cm).
