# Supplementary material for: Silver and Zinc Oxide Nanoparticles for Effective Aquaculture Wastewater Treatment
Source: Nanomaterials (Basel). 2025 Apr 5;15(7):559. doi: 10.3390/nano15070559 (PMC11990093; doi:10.3390/nano15070559)
Supplement: Supplementary file 1 [file nanomaterials-15-00559-s001.zip › nanomaterials-3575377-supplementary.pdf]

## Supplementary Data

# Silver and Zinc Oxide Nanoparticles for Effective Aquaculture Wastewater Treatment

**Mahmoud Abou-Okada [1,2], Mansour El-Matbouli [1] and Mona Saleh [1,*]**

[1] Division of Fish Health, University of Veterinary Medicine, 1210 Vienna, Austria; abouokada.mm@cu.edu.eg (M.A.-O.); mansour.el-matbouli@vetmeduni.ac.at (M.E.-M.)
[2] Aquatic Animal Medicine and Management, Faculty of Veterinary Medicine, Cairo University,
Giza 12211, Egypt
* Correspondence: mona.saleh@vetmeduni.ac.at

**Supplementary data Pages: 5**

**Supplementary data Tables: 1**

**Supplementary data Figures: 4**

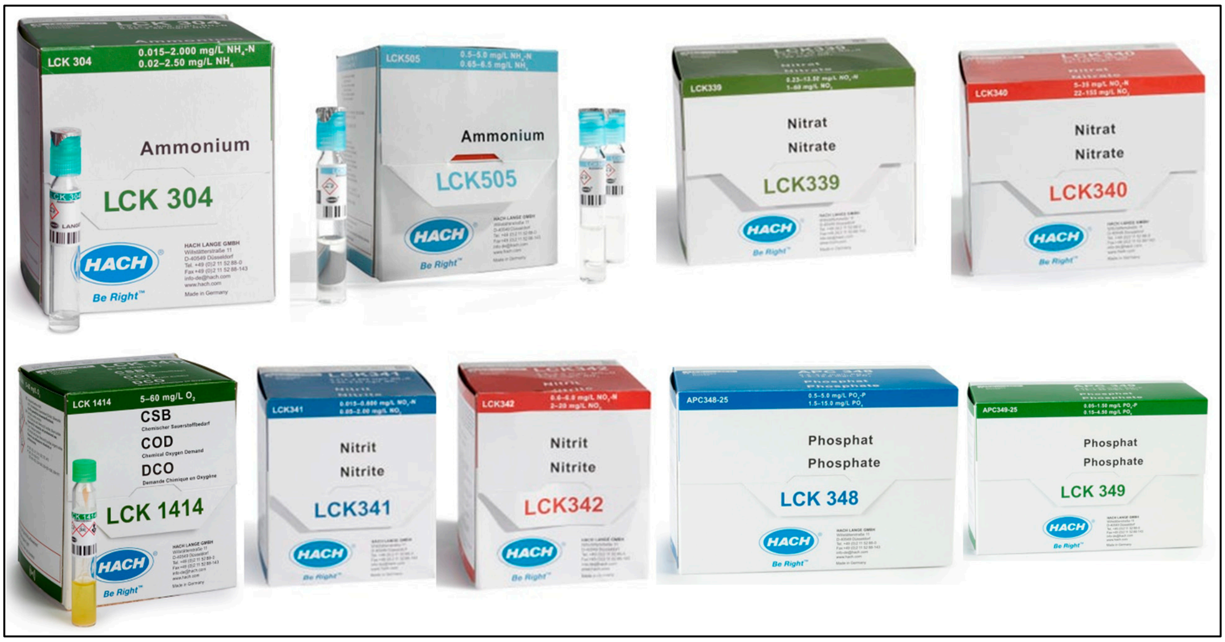


**Figure S1. Water quality kits (All these kits were obtained from Hach Lange, GmbH, Germany).**

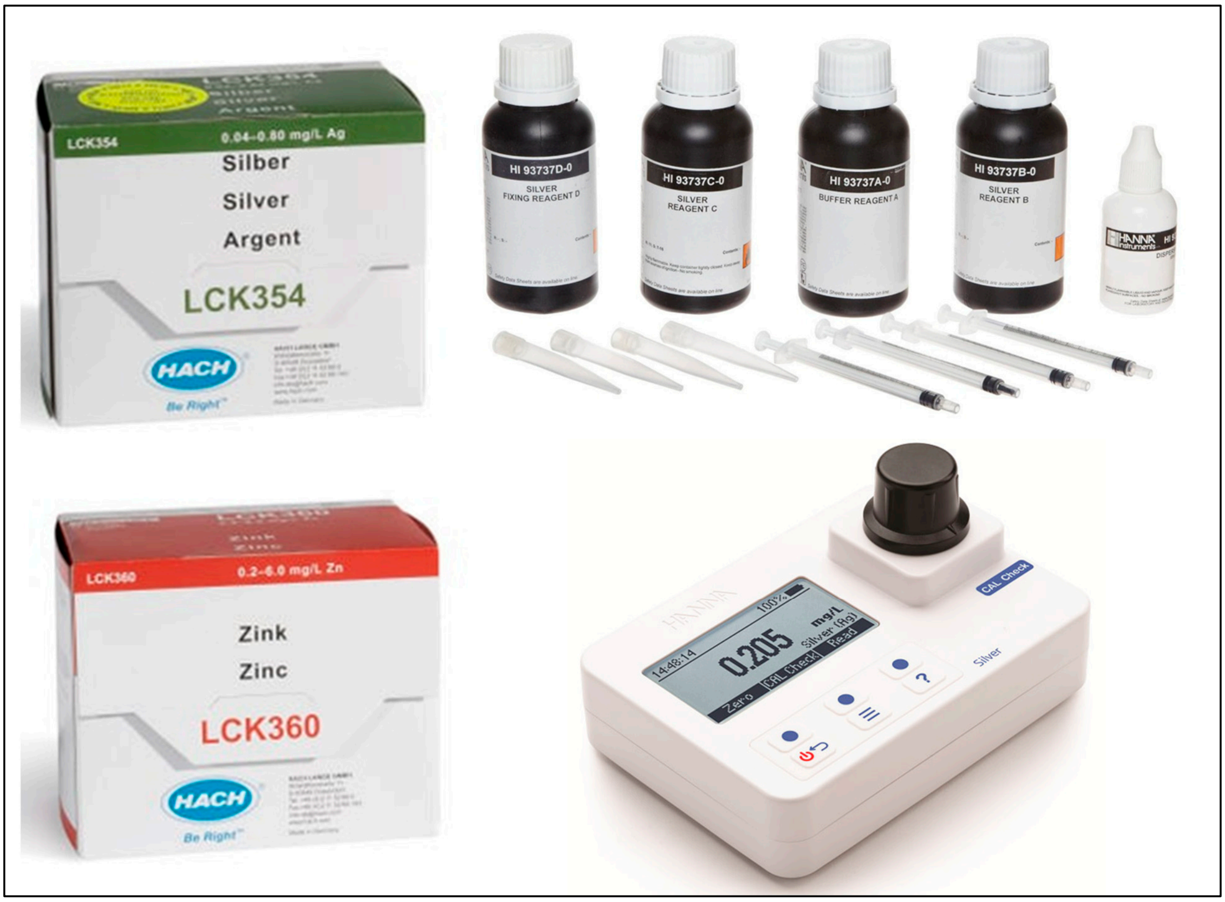


**Figure S2.** Silver and zinc kits (All these kits were obtained from Hach Lange, GmbH, Germany). HI-93737-01 reagents and HI-97737 Silver Photometer (Hanna Instruments, GmbH, Germany).

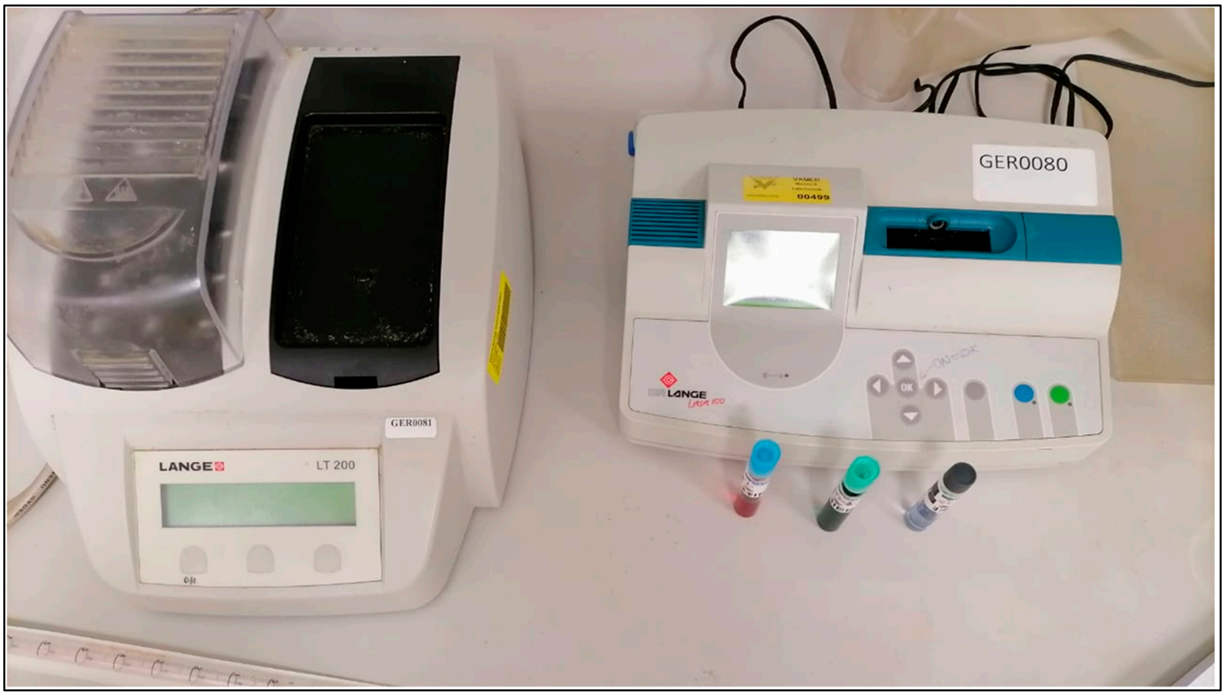


**Figure S3. DR1900 Hach Lange spectrophotometer and LT200 dry heater equipped with two blocks (Hach Lange, GmbH, Germany).**

**Table S1. ANOVA summary table for conductivity (EC, µS/cm)**

| Source of Variation | *df* | SS | MS | *F* | *p* | Effect size ($\omega^2$) |
|---|---|---|---|---|---|---|
| Treatment | 5 | 34.85 | 6.971 | 5.897 | 0.001 | 0.039 |
| Time | 3 | 549.4 | 183.1 | 154.9 | 0.001 | 0.736 |
| Interaction | 15 | 100.1 | 6.671 | 5.643 | 0.001 | 0.111 |
| Residual (Error) | 48 | 56.74 | 1.182 | | | |
| Total | 71 | 741.1 | | | | |

Note: df = degree of freedom, SS = Sum of squares, MS = Mean squares, $\omega^2$ = Omega squared.

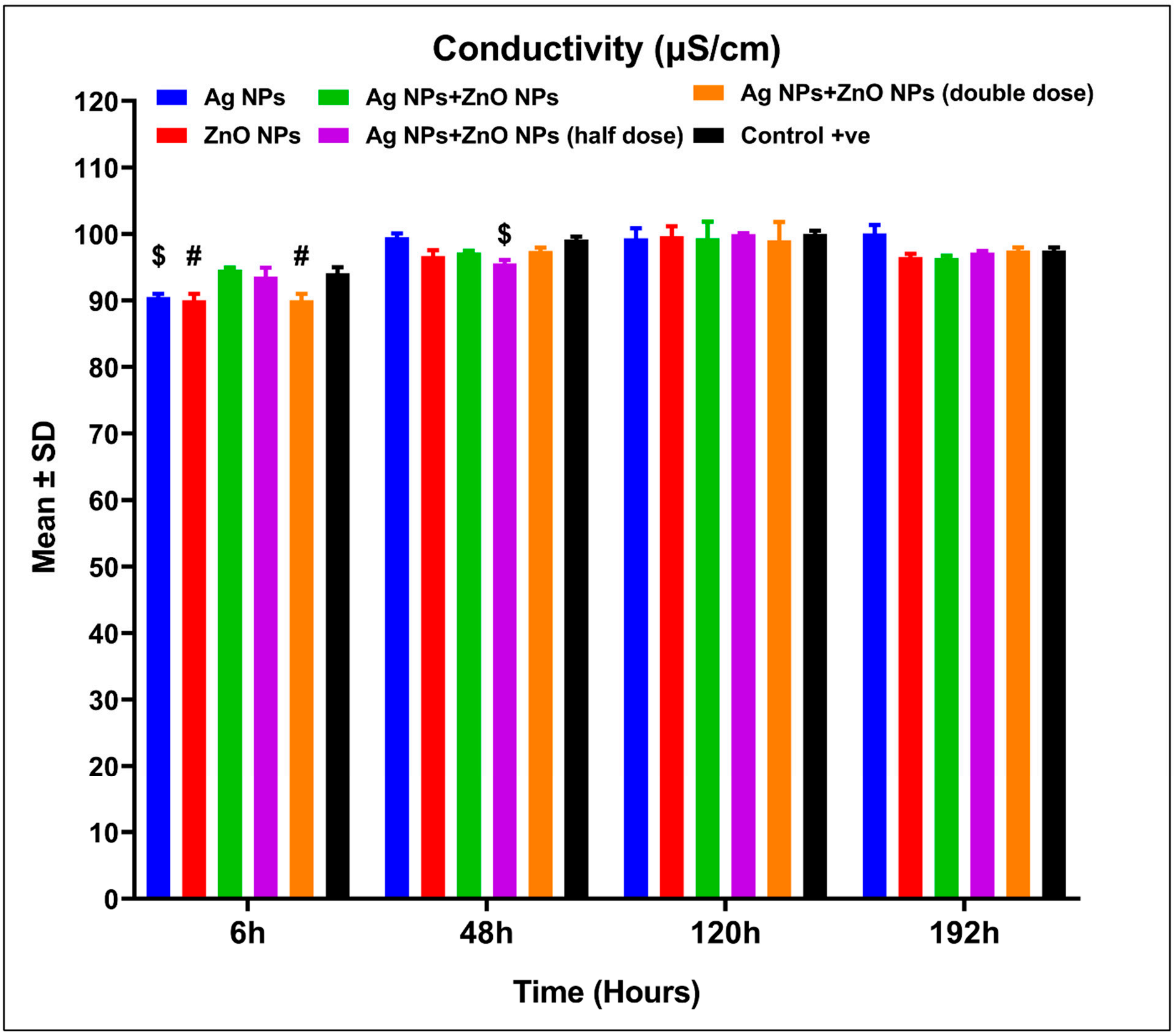


**Figure S4. The conductivity (EC, µS/cm) of aquaculture wastewater treated with nanoparticles was compared to that of untreated aquaculture wastewater (control +ve). The bars display the mean ± SD of the mean (n = 3). Statistically significant differences were observed at p < 0.05 (ANOVA, Tukey's post hoc). * (*p* < 0.05), $ (*p* < 0.01), and # (*p* < 0.001) when compared to the control group.**
